# Experiences and perceptions risk of health-care workers from coronavirus: A protocol for systematic review

**DOI:** 10.1097/MD.0000000000020308

**Published:** 2020-05-15

**Authors:** Peng Chen, Jiexin Lei, Fuchao Chen, Benhong Zhou

**Affiliations:** a Department of Pharmacy; b Department of Endocrinology, Renmin Hospital of Wuhan University, Wuhan; c Department of Pharmacy, Dongfeng Hospital, Hubei University of Medicine; Shiyan, Hubei, P.R. China.

**Keywords:** coronavirus, experiences and perceptions risk, healthcare workers, systematic review

## Abstract

**Background:**

Healthcare workers (HCWs) were at the frontline during the battle against coronavirus. Understanding and managing their fears and anxieties may hold lessons for handling future outbreaks. However, the experiences and perceptions risk of HCWs from coronavirus still remains to be controversial. Thus, the objective of this review is to identify, appraise, and synthesize available evidence related to the experiences and perceptions of risk of HCWs from coronavirus.

**Methods:**

The studies were gathered from PubMed, Cochrane Library, EMBASE, CBMdisc, CNKI, WKSP, CSJFT, Google Scholar, and PsycINFO, along with several sources of gray literature. The retrieval of full-text studies, data extraction, and quality assessment of the included studies will be independently conducted by 2 reviewers. The meta-aggregative will be used for findings pooling and a summary of ConQual findings tables will be presented in future.

**Results:**

This study will be submitted to a peer-reviewed journal for publication.

**Conclusion:**

The literature will provide a high-quality analysis of the current evidence to assess the experiences and perceptions risk of health-care workers from coronavirus.

**Registration information:**

CRD42020170388.

## 1 Introduction

Coronaviruses are enveloped nonsegmented, single-stranded, positive-sense RNA viruses classified under the genus Coronavirus within the family Coronaviridae and the order Nidovirales.^[1]^ The coronaviruses are host-specific and can infect humans as well as a variety of different other non-human mammals, resulting in seemingly related diverse clinical syndromes.^[2]^ Although the human coronaviruses are usually mild and consistent with the common cold but the epidemics of the 2 betacoronaviruses, severe acute respiratory syndrome coronavirus (SARS-CoV)2–4 and Middle East respiratory syndrome coronavirus, has caused more than 10,000 deaths in the past 2 decades has demonstrated, which approximately 50% rooted in SARS-CoV and Middle East respiratory syndrome coronavirus.^[3,4]^ In late December 2019, an ongoing outbreak of pneumonia associated with a novel coronavirus designated 2019-novel coronavirus (2019-nCoV) was reported in Wuhan city, Hubei province. Thus far, confirmed cases in Hubei has now reached 1052 including health-care workers, with 129 infected patients in serious condition, while the death toll climbed to 52, and several exported cases have also been confirmed in other provinces in China, and in Japan, South Korea, Thailand, and the USA.^[5,6]^ These data presents a timely reminder that despite the significant medical gains of the last century, the danger posed by emerging infectious diseases has become even greater in our increasingly interconnected world.^[7]^

Healthcare workers (HCWs) were at the frontline during the battle against emerging infectious diseases to save lives while endangering their own.^[8]^ It has been reported that HCWs were the most at risk, accounting for 21% to 24% of all cases worldwide and Canada had the highest proportion of HCWs affected (43%), with China coming in a close second (40.8%).^[9]^ The current outbreak of the 2019-nCoV has caused about 25 cases of infected HCWs and 1 death of HCW in beginning of outbreak.^[10]^ Despite being hailed as heroes for their courage in facing a deadly infectious disease for which there was no known effective treatment, many HCWs endured social stigmatization and in some cases even faced ostracism from their own families.^[11,12]^ These features pose some problems for health authorities and health-care professionals: How did they feel? What went through their minds? How were their lives impacted?

The HCWs issues surrounding the previous acute infectious diseases (AIFDs), such as the influenza pandemic in the last century, have never been fully solved or partially addressed.^[13,14]^ To address the antecedents of HCWs issues, understanding the components that influence their risk perception and experiences towards different AIFDs is considered a useful approach.^[15]^ Individuals’ risk appraisal and perception, which depicts the perceived susceptibility to a threat to health, is considered the essential constituent that mediates attitude change and navigates decision making. Indeed, previous research has established that HCWs’ compliance with precautionary measures is primarily related to their personal anticipation of the level of risk for AIFDs.^[16]^ With the resurgence of emerging acute respiratory infectious diseases such as SARS and 2019-nCoV in Wuhan, research investigating how HCWs’ experiences and perceptions affect their clinical decision making towards their exposure is more than ever pertinent.^[10]^ Panic of clinicians can be better avoided by candid acknowledgment of the risks and timely implementation of simple protective measures based on development of evidenced-based guidelines.^[18]^ This will lead to improve the protection of HCWs and enhance the communication outcomes of safety measures, so that they can be better prepared for the next battle.

A preliminary search was performed in January 2020, which included the Cochrane Library, JBI Database of Systematic Reviews, PROSPERO, the Centre for Reviews, and web of science. Several relevant papers has been observed in this search, including 2 literature reviews, that focused solely on HCWs’ perceptions of risk from SARS.^[19,20]^ However, to our knowledge, there no systematic review on the topic has been conducted to date, thereby providing a strong rationale for this review. Therefore, the author will collect high-quality research literature in authoritative databases at home and abroad and to address this gap and identify, appraise, and synthesize all available evidence related to the experiences and perceptions risk of health-care workers from coronavirus.

### 1.1 Review question

What are the risk of perceptions and experiences on personal and work life of HCWs during the coronaviruses epidemic of exposure to coronaviruses and the factors that influenced this perception and experiences risk.

## 2 Methods

### 2.1 Study registration

The protocol has been registered on the International Prospective Register of Systematic Reviews (PROSPERO), the registration number is CRD42020170388 (available from https://www.crd.york.ac.uk/prospero/display_record.php?ID=CRD42020170388). The content followed preferred reporting items for systematic review and meta-analysis protocols.^[22]^

### 2.2 Inclusion criteria

#### 2.2.1 Participants

Only published articles enrolling all HCWs from coronavirus, working in urban and rural of healthcare settings worldwide. The gender and age of participants will not be limited.

#### 2.2.2 Phenomena of interest

The studies that inspect the risk of perceptions and experiences of HCWs from coronavirus will be all considered in this qualitative review. The term “experiences and perceptions” consisted of all factors impact on the health issues of HCWs from coronavirus. Coronavirus diagnosis was in accordance with the World Health Organization: Clinical management of severe acute respiratory infection when nCoV infection is suspected: Interim Guidance.

#### 2.2.3 Types of studies

This review involves experiences and perceptions risk of health-care workers in relation to the topic. In order to answer the question of this review, mixed methods studies, where qualitative and quantitative methods are used together, but not limited to, ethnography, case studies, action research and grounded theory, and phenomenology cannot be ignored. Furthermore, a comprehensive search strategy will be used to obtain studies published in black or gray literature. No language limits were set on the database searches. There were no language restrictions for inclusion in this review. And there were no resources of translation for our review team members.

#### 2.2.4 Outcomes

##### 2.2.4.1 Primary outcome

The primary outcome is that perception of risk of exposure to coronavirus.

##### 2.2.4.2 Secondary outcome

The secondary outcome is the impact of events scale which measures the intrusive and avoidance items made by people during stressful life events.^[7]^ The responses were recorded on a 6-point Likert scale (1 strongly disagree, 6 strongly agree) with scores of 1 to 3 taken as indicative of negative response, and 4 to 6 as positive response. The scores obtained for the intrusion avoidance items in the IES were divided into “high” or “low” scores, using the median score as the cut-off point.

### 2.3 Search strategy

Both published and unpublished studies are available for this systematic review. Two reviewers (Chen P and Chen FC) independently searched the medical literature for relevant clinical trials using the electronic databases of PubMed, Cochrane Library, EMBASE, CBMdisc, CNKI, WKSP, CSJFT, Google Scholar, and PsycINFO until February, 2020. This was supplemented by searching the reference lists of all retrieved studies, review articles, abstracts, and conference reports. The keywords and index terms used in this search were all adapted for each included information source. Used PubMed as an example, the search strategy for PubMed is summarized in Table 1. There were no language restrictions.

**Table 1:**
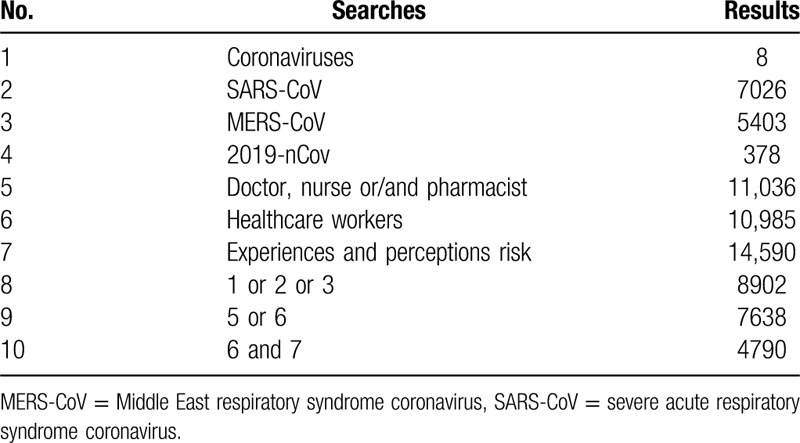
Pubmed search strategies.

### 2.4 Information sources

We will search electronic academic databases and relevant public health websites for potentially identify relevant records from published and un-published. If necessary, we will contact with the study authors. The databases to be searched include: PubMed, Cochrane Library, EMBASE, CBMdisc, CNKI, WKSP, CSJFT, Google Scholar, and PsycINFO. The search for unpublished or gray literature will include: ProQuest Dissertations and Theses, HSRProj, Grey Matters, Web of Science Conference Proceedings, OpenGrey, Lenus, RIAN, and Grey Literature Report (U.S. context). The key terms that will inform the development of strategies for each database are derived from MEDLINE and will be revised and combined with free text terms before the full search is conducted in the relevant databases.

### 2.5 Study selection

The extraction of the synthesized results uploaded to and merged using Endnote X7 software (Clarivate Analytics, PA). If duplicate publications are identified, they will be removed from the list. The potential literatures were considered eligible by reading the title and abstract performed by 2 independent reviewers, according to the established inclusion criteria. Potentially relevant studies will be retrieved in full and their citation details imported into the JBI System for the Unified Management, Assessment and Review of Information (JBI SUMARI) (Joanna Briggs Institute, Adelaide, Australia). For the full-text articles analysis, 2 independent reviewers will assess in detail against the inclusion criteria to excluded the studies which did not meet the inclusion criteria were also excluded, and reasons for exclusion of full-text studies will be recorded and reported in the systematic review. Any disagreements that arise between the reviewers will be resolved by discussion at each stage.

### 2.6 Assessment of methodological quality

The standardized critical appraisal instrument from the JBI SUMARI will be used to evaluated the methodological quality of the included studies.^[21]^ The primary authors of the reports and papers will be contacted where necessary to request missing or additional data or re-analysis. Any disagreements that arise between the reviewers will be resolved via discussion, or with a third reviewer. All relevant studies will be included in the review regardless of methodological quality and design and undergo data extraction and synthesis (where possible). The critical appraisal results will be presented in narrative form and in a table.

### 2.7 Data extraction

The standardized data extraction tool from JBI SUMARI will be used to extract qualitative data from papers included in the review.^[21]^ We extracted from the studies in the following information: the first author, year of publication, populations enrolled in the study, context, culture, geographical location, study methods, and the phenomena of interest relevant to the review objective (ie, What are the risk of perceptions and experiences on personal and work life of HCWs during the coronaviruses epidemic of exposure to coronaviruses and the factors that influenced this perception and experiences risk). The extracted findings from each paper will be examined for congruency and agreement by the primary and secondary reviewers.

### 2.8 Data synthesis

Qualitative research findings will be pooled using JBI SUMARI with the meta-aggregation approach.^[21,23]^ This will involve the aggregation or synthesis of findings to generate a set of statements that represent that aggregation, through assembling the findings and categorizing these findings based on similarity in meaning. These categories will then be subjected to a synthesis to produce a single comprehensive set of synthesized findings that can be used as a basis for evidence-based practice. Where textual pooling is not possible, the findings will be presented in narrative form. The findings will be interpreted and compared in accordance with different settings where studies were based.

### 2.9 Assessing confidence in the findings

The final synthesized findings will be graded according to the ConQual approach for establishing confidence in the output of qualitative research synthesis and presented in a Summary of Findings.^[21,23]^ The Summary of Findings includes the major elements of the review and details on how the ConQual score is developed. Included in this table are the title, population, phenomena of interest, and context for the specific review. Each synthesized finding from the review will then be presented along with the type of research informing it, a score for dependability, credibility, and the overall ConQual score.

## 3 Discussion

HCWs are often exposed to a variety of occupational hazards within their workplaces, in particular, infectious diseases such coronavirus, some of which may cause death.^[17]^ Learning about the fears, anxieties, and reactions of HCWs during the coronavirus (especially for 2019-nCoV) epidemic may hold important lessons for the handling of future epidemics and acts of bioterrorism.^[5,6,10]^ Our data show that the HCWs in this study have similar concerns to previous research on HCW's perceptions of risk from SARS and other emerging acute respiratory infectious diseases in that these HCWs were concerned about risks to their personal health (from patients, from colleagues and visitors to the organization).^[24]^ They were also concerned about the health risks that their employment as a HCW might cause to others, in particular those more vulnerable such as the elderly.^[25]^ Finally, the study findings clearly indicate that although participants perceived themselves to be at risk of infection, all of them were accepting of these risks as they saw it to be part of their professional obligation. HCWs surveyed in studies about their risk perceptions during coronavirus and possible pandemic influenza pandemics likewise expressed the same perceptions and willingness to serve in such pandemics.

As the systematic review is based on our previously published research, there are undeniable methodological differences among the included studies. Furthermore, the quality level and reliability of the final results in this review determines as the quality of the included studies. The review will be conducted again if e necessary included studies are met, and to ensure that the provided information can be fully helpful for clinicians and patients, all operating procedures will be performed in accordance of JBI methodology for systematic reviews of qualitative evidence.

## Author contributions

**Conceptualization:** Peng Chen.

**Data curation:** Peng Chen.

**Formal analysis:** Peng Chen.

**Investigation:** Peng Chen.

**Methodology:** Peng Chen.

**Project administration:** Peng Chen, Zhou Ben Hong.

**Resources:** Peng Chen.

**Software:** Peng Chen, Fuchao Chen, Jiexin Lei.

**Supervision:** Peng Chen.

**Validation:** Peng Chen.

**Visualization:** Peng Chen.

**Writing – original draft:** Peng Chen.

**Writing – review and editing:** Peng Chen.

Zhou Ben Hong orcid: 0000-0002-6129-0182.
